# Comparison of 6-[^18^F]FDOPA PET with Nigrosome 1 detection in patients with parkinsonism

**DOI:** 10.1186/s13550-021-00758-x

**Published:** 2021-02-15

**Authors:** Enrico Michler, Daniel Kaiser, Kiriaki Eleftheriadou, Björn Falkenburger, Jörg Kotzerke, Sebastian Hoberück

**Affiliations:** 1 Department of Nuclear Medicine, Faculty of Medicine and University Hospital Carl Gustav Carus, TU Dresden, Dresden, Germany; 2 Department of Neuroradiology, Faculty of Medicine and University Hospital Carl Gustav Carus, TU Dresden, Dresden, Germany; 3 Department of Neurology, Faculty of Medicine and University Hospital Carl Gustav Carus, TU Dresden, Dresden, Germany; 4 German Center for Neurodegenerative Diseases (DZNE), Dresden, Germany

**Keywords:** Nigrosome 1, 6-[^18^F]FDOPA, PET, Parkinson’s disease, Parkinsonism

## Abstract

**Background:**

The functional 6-[^18^F]FDOPA positron emission tomography (PET) can be a helpful tool in differentiating parkinsonism with dopaminergic deficiency from clinically similar differential diagnoses. Furthermore, in T2*/susceptibility-weighted imaging (SWI) magnetic resonance imaging (MRI) sequences the structural integrity of the Nigrosome 1 (N1) can be assessed by checking the presence of the swallow tail sign (STS). We therefore retrospectively compared the performance of the 6-[^18^F]FDOPA PET with the N1 detection in patients suspected with parkinsonian diseases. Forty-three consecutive patients (m: 23, f: 20, mean age: 63 ± 12 years) were included in the study. They underwent clinically indicated 6-[^18^F]FDOPA PET/MRI scans as part of their neurological evaluation of uncertain parkinsonian syndromes. Visual and semi-quantitative PET imaging results were statistically compared with visual N1 assessment on 3 T SWI. As the gold standard, we defined the clinical diagnosis at the last follow-up, which included idiopathic Parkinson syndrome (IPS; *n* = 18), atypical parkinsonian syndromes (APS; *n* = 9) and other neurological diseases without dopaminergic deficit (*n* = 16).

**Results:**

Thirty-five of 43 patients (81%, Kappa 0.611) had corresponding results in 6-[^18^F]FDOPA PET and SWI. Seven of the remaining 8 patients were correctly diagnosed by 6-[^18^F]FDOPA PET alone. Sensitivity, specificity and accuracy for 6-[^18^F]FDOPA and N1 imaging were 93%, 94%, 93% and 82%, 75%, 79%, respectively.

**Conclusions:**

6-[^18^F]FDOPA PET and Nigrosome 1 evaluation had an overall good intermodality agreement. Diagnostic agreement was very good in cases of clinically suspected idiopathic Parkinson syndrome and fair in atypical parkinsonian syndromes, but poor in patients with non-parkinsonian disorders. 6-[^18^F]FDOPA PET showed higher sensitivity, specificity and accuracy in discriminating parkinsonian syndromes from non-parkinsonian disorders than the N1 evaluation. In summary, the additional benefit of N1 assessment in patients with APS or parkinsonism without dopaminergic deficit needs to be proven by prospective studies.

## Background

Idiopathic Parkinson syndrome (IPS) is a sporadic neurodegenerative disease, in which pathological intracellular deposition of a-synuclein leads to diminishing motor and non-motor functions [1]. After onset in the brain stem, the disease progresses into midbrain’s substantia nigra, resulting in dopaminergic cell death [2]. Besides IPS, further neurodegenerative disorders show a dopaminergic deficit. These are called atypical parkinsonian syndromes (APS): progressive supranuclear palsy (PSP), multiple system atrophy (MSA), corticobasal degeneration (CBD) and dementia with Lewy Bodies (LBD).

In general, the aforementioned Parkinson syndromes (PS) are diagnosed by clinical examination. However, in uncertain cases, extended diagnostic imaging evaluation is needed to distinguish PS from parkinsonism without dopaminergic deficit, such as vascular encephalopathy, medication side effects or essential tremor. On such matters, ^123^I-FP-CIT (DaTSCAN) serves as a well-established radiotracer for presynaptic dopamine imaging [3]. In the last two decades, 6-[^18^F]FDOPA positron emission tomography (PET) emerged as a very sensitive neuroimaging tool for detection of nigrostriatal dopaminergic degeneration [4]. The combination of 6-[^18^F]FDOPA PET with magnetic resonance imaging (MRI) provides assessment of both functional and structural integrity of the striatum and other brain regions, thus enhancing the informative value of each component. Recently, another structure gained attention as a potential biomarker of IPS: the Nigrosome 1 (N1), a neuromelanin-rich cluster of the substantia nigra pars compacta [5]. In healthy individuals, N1 can be visualized on high-resolution susceptibility-weighted imaging (SWI) MRI as a linear high signal structure that divides the posterior third of the substantia nigra into a configuration that appears like a swallow tail. The absence of N1 and swallow tail sign showed a high accuracy in distinguishing patients with IPS from healthy controls and a good inter-rater reliability [6, 7].

The role of N1 assessment in distinguishing patients with IPS and APS from movement disorders without dopaminergic deficit remains unclear. As 3 T-MRI platforms are widely available and cheaper than PET imaging on a daily basis, N1 evaluation may challenge the predominance of nuclear medicine techniques in this special field. We therefore aimed to compare metabolical und morphological results of 6-[^18^F]FDOPA PET/MRI in patients with PS, according to the clinical gold standard.

## Methods

### Patients and neurological evaluation

We retrospectively analyzed data of patients who received a 6-[^18^F]FDOPA PET/MR scan between 01/2014 and 05/2019 in addition to their neurological evaluation. Inclusion criteria for this study were evaluable PET and MRI data, at least six months of clinical follow-up after PET/MR scan, and a definitive diagnosis of PS or Non-PS by a specialist of the neurological department of our university hospital. Clinical diagnoses were made according to the 2015 Movement Disorder Society (MDS) criteria for IPS [8]. In cases of APS, diagnoses were based on current international consensus criteria for each individual disease entity [9–11]. The same applies to the clinical diagnoses of Non-PS patients [12–15]. A typical interval between clinical assessment and diagnosis was 0–3 months. Inpatients were seen, referred to imaging and diagnosis based on clinical symptoms and imaging results. Outpatients were seen, referred for imaging and the results discussed on the next visit, which was usually 3 months later. Exclusion criteria were missing of a definitive clinical diagnosis, less than 6 months or missing of clinical follow-up, missing of SWI-sequences, and a non-evaluable N1 (f.e. due to patient movement). The local institutional ethics committee approved the study (ID number: BO-EK-27012020). Clinical, demographic and technical data were obtained after inclusion into the study (patient age at scan, sex, duration of disease at scan, administered 6-[^18^F]FDOPA activity, clinical diagnosis prior to scan, final clinical diagnosis after follow-up).

### Image acquisition

The PET was performed on a time-of-flight sequential PET/MR system (Philips Ingenuity TF 3 T, Philips Medical Systems, Best, Netherlands). Any drugs that may interact with 6-[^18^F]FDOPA, such as dopamine agonists, dopamine reuptake inhibitors, dopamine releasing agents, peripheral catechol-omethyltransferase inhibitors or monoamine oxidase inhibitors, were discontinued according to their corresponding half-lives before 6-[^18^F]FDOPA injection. Patients were premedicated with Carbidopa, 100 mg orally 1 h prior to injection of 215.16 ± 18.31 MBq 6-[^18^F]FDOPA [16].

Scanning involved the positioning in a dedicated MR eight-channel head coil. The scanning protocol started with a nondiagnostic T1-weighted fast field echo scan for attenuation correction.

PET images were acquired over 15 min in list mode divided into 3 frames of 5 min each, starting either 60 min or 75 min p.i. These time intervals were chosen from existing prepublished reference values obtained from healthy subjects and PD patients [17].

Susceptibility weighted imaging (SWI) high-resolution sequence (TR/TE 15/25 echo train length 1, Flip angle 10°, number of slices: 200, voxel size 1 × 1 × 1 mm, spacing between slices 0.5 mm, scan duration 6 min 23 s) was aligned parallel to the anterior commissure-posterior commissure line.

Qualitative and quantitative evaluation was performed on the attenuation corrected data set. Neither the device nor the acquisition protocol changed among all PET/MR examinations included in this study.

### Image analysis

Data evaluation was performed using the software package ROVER (abx, Radeberg, Germany). Scans were checked for head movement, and motion correction was performed if necessary.

**Fig. 1:**
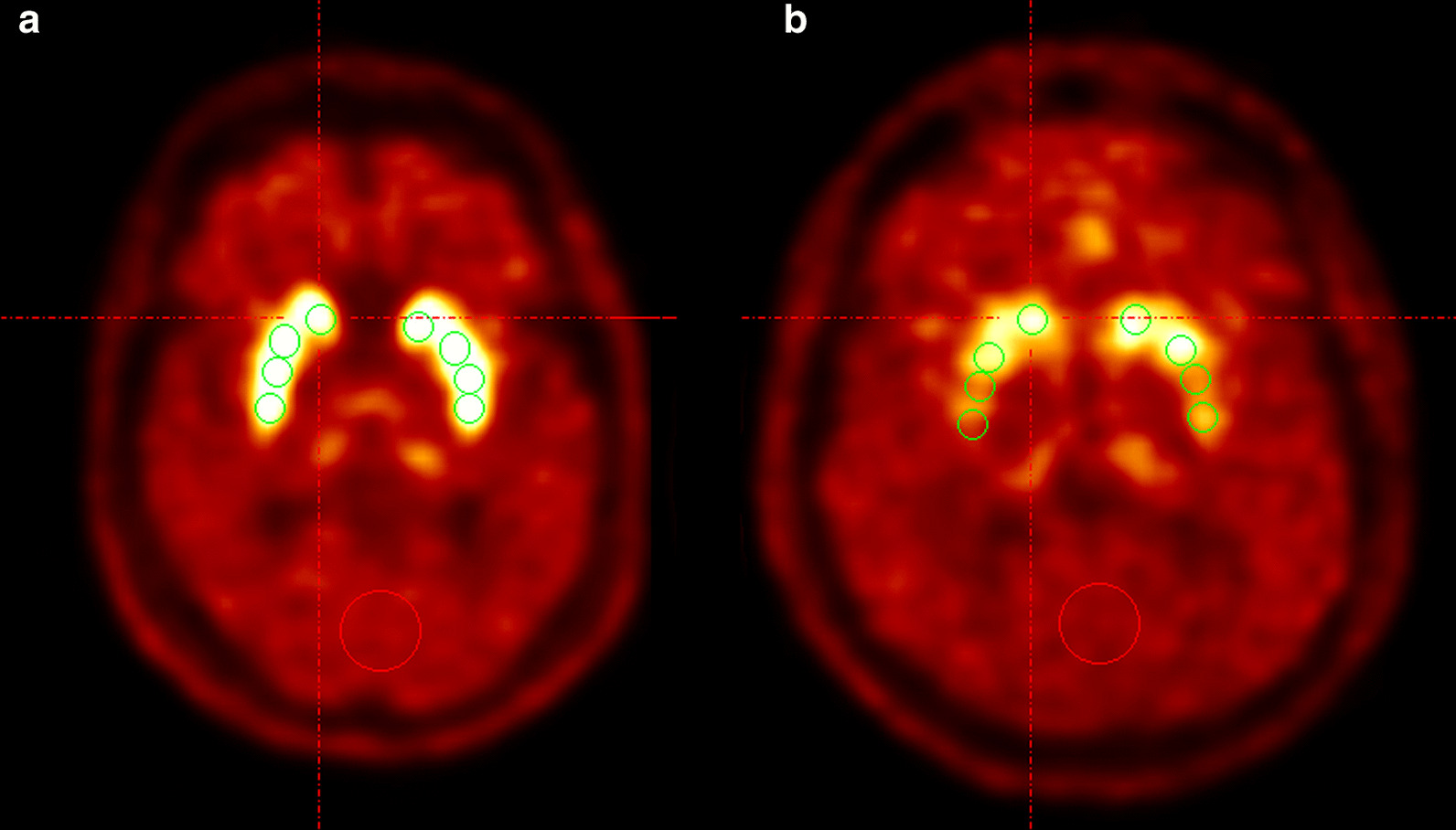
Evaluation of presynaptic dopamine metabolism in 6-[^18^F]FDOPA PET. Tracer uptake assessment in the bilateral striatum shown in axial plane imaging (**a**, **b**). Spheric VOIs (green circles) were placed in each caput of the caudate nucleus and the anterior, middle and posterior part of both putamina. A single reference sphere was positioned in the occipital cortex (red circles). The left image shows physiologic 6-[^18^F]FDOPA uptake in the striatum (**a**). The right image shows severely reduced uptake in the middle and posterior putamen on both sides, representing a dopaminergic deficit (**b**)

For 6-[^18^F]FDOPA uptake quantitation, a set of predefined [17] spherical volumes of interest (VOI) was used: One sphere of 0.3 ml was positioned in each caput of the caudate nucleus. Three spheres of each 0.3 ml were placed in anterior, middle and posterior part of the left and right putamen, as shown in Fig. 1. One reference sphere of 5 ml was positioned in the occipital cortex. The ratios were calculated by division of the value of the Caput nucleus caudatus, respectively, the mean of the three VOIs of the putamen with the reference value. The rostro-caudal gradient analogously was determined by comparison of the values of the three putaminal portions on both sides. All VOIs were positioned consensually by two (SH, EM) experienced Nuclear Medicine specialists.

These data were compared to ranges of historic reference values, derived from mean ± 2 standard deviations of a group of 9 healthy controls (5 female, 4 male, mean age 63 ± 8 years), investigated on a dedicated PET scanner (ECAT EXACT HR + , Siemens/CTI) with identical PET scanning protocol and identical VOI evaluation [17].

According to calculated data and visual image impressions, the PET results were categorized as follows: normal (no presynaptic nigrostriatal deficit) and abnormal (presynaptic nigrostriatal deficit). There was no non-diagnostic case.

**Fig. 2:**
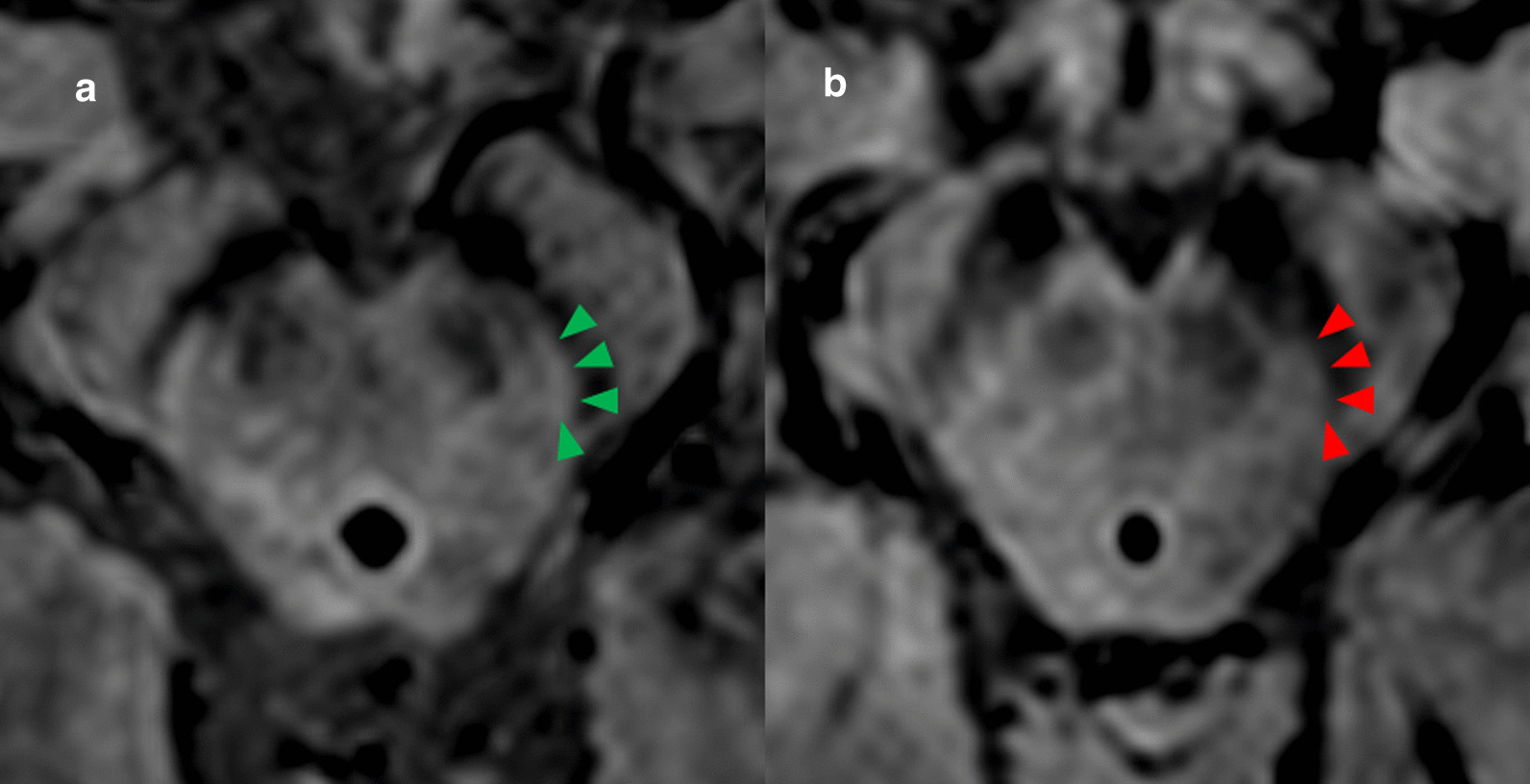
Evaluation of Nigrosome 1 in high-resolution MRI. Nigrosome 1 assessment of the substantia nigra in axial plane susceptibility weighted imaging MRI of the midbrain. Green arrows highlight the presence of Nigrosome 1 in a 62-year-old man (**a**). Red arrows highlight the absence of Nigrosome 1 in substantia nigra in a 65-year-old woman (**b**)

Visually assessment of SWI scans for the presence or absence of Nigrosome 1 was performed as described before ([7], Fig. 2). The axial scans were reviewed with local picture archiving and communication system (IMPAX EE, AGFA HealthCare, Bonn, Germany) on high-resolution widescreen LCD displays. In detail, we performed a blinded rating of the N1 for each hemi-mesencephalon at the level of the caudal posterior substantia nigra. The five-level rating scale consisted of the items: (1) N1 present, (2) N1 possibly present, (3) possibly absent, (4) N1 absent and (5) non-evaluable, whereas motion artifacts were the main reason for non-evaluable score. According to N1 rating, we divided the scans in normal (N1 present bilaterally or unilateral N1 (possibly) present and contralateral possibly present) and abnormal (N1 (possibly) absent bilateral or unilateral). Non-diagnostic cases (N1 possibly present and possibly absent other side) were excluded from the study, as described above.

### Statistical analysis

Data analysis was conducted in SPSS Statistics Version 25 (IBM, Armonk, New York). Clinical characteristics were compared by chi-square test, Fisher’s exact test and t-test, as suitable and mentioned in the text. For multiple group comparison, ANOVA was used. Sensitivity, specificity, positive and negative predictive value and accuracy were calculated for each modality. Significance was determined as *p* < 0.05. Intermodality agreement was calculated by Cohen’s Kappa and categorized as follows [18]: poor (< 0.40), fair to good (0.41–0.75) and excellent (0.76–1.0).

## Results

### Patients and clinical diagnosis

**Fig. 3:**
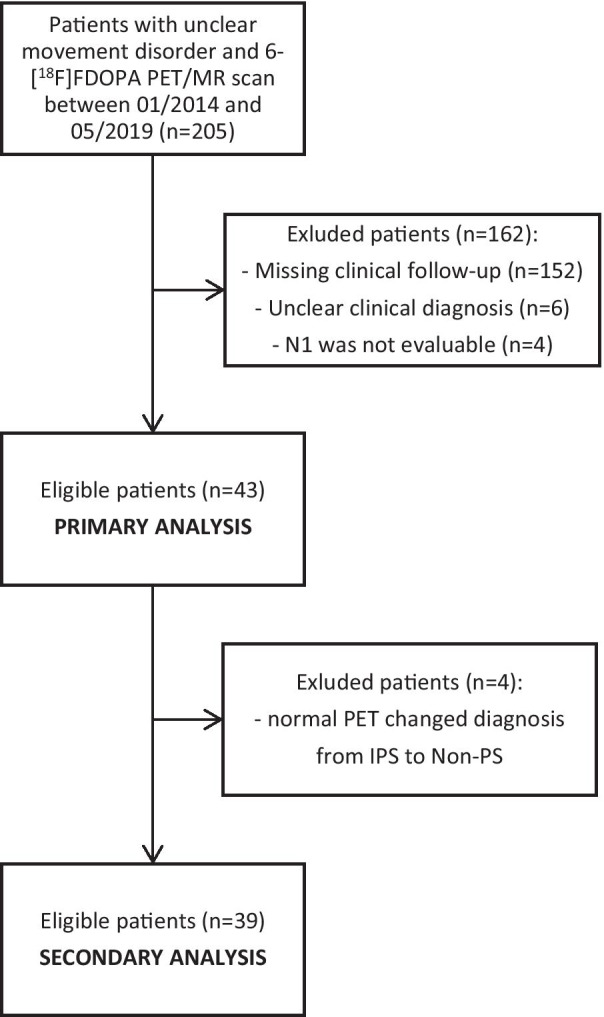
Patient flowchart of the study

Between 01/2015 and 05/2019, we performed 6-[^18^F]FDOPA PET/MRI in 255 consecutive patients with clinically uncertain movement disorders. The study flowchart is illustrated in Fig. 3. Clinical follow-up was incomplete or missing in 152 patients, so they were excluded from the study. Six patients were excluded because their clinical diagnoses were still unclear in the follow-up examination. Another four of 47 remaining patients had to be excluded, because N1 could not be evaluated (due to patient movement). Eventually, 43 patients were included into the study for primary analysis (23 male, mean age: 63.30 ± 12.22 years). Basic demographic characteristics are shown in Table 1.

**Table 1:** Patient characteristics and statistical comparison of the PS group and the Non-PS group

Characteristics	All patients	PS	Non-PS	*p* value
No. of male/female patients (% of total)	23 (54%)/20 (46%)	15 (56%)/12 (44%)	8 (50%)/8 (50%)	0.732
Age (years) mean ± SD (range)	63.30 ± 12.22 (20–82)	63.19 ± 10.46 (38–82)	63.50 ± 15.13 (20–81)	0.936
Duration of disease (years) mean ± SD (range)	3.23 ± 2.89 (1–10)	2.56 ± 1.97 (1–10)	4.38 ± 3.79 (1–10)	0.044
Activity (MBq) mean ± SD	215.16 ± 18.31	215.63 ± 17.30	214.38 ± 20.45	0.831

*PS* Parkinson syndrome, *SD* standard deviation

Twenty-seven of 43 evaluable patients were clinically diagnosed with idiopathic or atypical parkinsonian syndrome (15 male, mean age: 63.19 ± 10.46 years, mean duration of disease prior to PET/MR imaging: 2.56 ± 1.97 years). Eighteen of those patients had an IPS (12 male, mean age: 62.00 ± 12.31 years). Nine patients had an APS (3 male, mean age: 65.56 ± 4.90 years), as shown in Table 2. The APS group consisted of six subjects with progressive supranuclear palsy, two subjects with corticobasal degeneration and one subject with multiple system atrophy. The remaining 16 patients (8 male, mean age 63.50 ± 15.13 years, mean duration of disease: 4.38 ± 3.79 years) were diagnosed with other neurological diseases than IPS or APS. Diagnoses were as follows: six subjects with essential tremor (ET), two subjects with drug-induced parkinsonism (DIP), three subjects with vascular parkinsonism (VP) and five subjects with a diagnosis of restless legs syndrome (RLS), ataxia, motor neuron disease (MND), Alzheimer’s disease (AD) or Huntington’s disease (HD), respectively. Gender distribution and age did not vary between both of the PS group and the Non-PS group, neither did the administered 6-[^18^F]FDOPA activity. Mean duration of disease prior to PET/MR imaging was significantly shorter in PS patients than in those with other movement disorders (*p* = 0.044). A wide range of disease duration was observed in both groups (1–10 years).

**Table 2:** Clinical diagnoses and their corresponding imaging results

Clinical gold standard diagnosis	Summed [*n*]	6-[^18^F]FDOPA abnormal [*n*, %]	N1 abnormal [*n*, %]
**IPS**	18	18 (100%)	17 (94%)
**APS**	9	7 (77%)	5 (55%)
Progressive supranuclear palsy	6	5 (83%)	3 (50%)
Corticobasal degeneration	2	1 (50%)	1 (50%)
Multiple system atrophy	1	1 (100%)	1 (100%)
**Non-parkinsonian movement disorders**	16	1 (6%)	4 (25%)
Essential tremor	6	0 (0%)	2 (33%)
Vascular parkinsonism	3	0 (0%)	1 (33%)
Drug-induced parkinsonism	2	0 (0%)	0 (0%)
Restless legs syndrome	1	1 (100%)	0 (0%)
Ataxia	1	0 (0%)	0 (0%)
Motor neuron disease	1	0 (0%)	1 (100%)
Alzheimer’s disease	1	0 (0%)	0 (0%)
Huntington’s disease	1	0 (0%)	0 (0%)

*IPS* idiopathic Parkinson syndrome, *APS* atypical parkinsonian syndrome

### Imaging

6-[^18^F]FDOPA PET imaging compiled positive (“abnormal”) results in 26 cases, suggestive for PS, and 17 negative (“normal”) results, suggestive for non-parkinsonian movement disorders. N1 assessment had 26 abnormal results and 17 normal results, as well. While total numbers of positive and negative test results were similar in both modalities, 6-[^18^F]FDOPA and N1 imaging corresponded only in 35 of all 43 cases (80%).

The *k* value of intermodality agreement was 0.611 in all patients (*p* < 0.001) and 0.521 in patients with PS (*p* = 0.002), which suggests a fair to good agreement between 6-[^18^F]FDOPA and N1 results in those groups. However, *k* value was 0.111 in patients without dopaminergic deficit (*p* = 0.551), which is a poor agreement.

Constellations of matching and mismatching results are shown in Table 3. Twenty-two cases were consistently classified as pathologic by both modalities—all of them had a clinical diagnosis of PS (17 patients with IPS and 5 patients with APS). Another 13 cases had normal PET and N1 results, but two of them were nonetheless clinically diagnosed with a PS (one patient with CBD and one patient with PSP). Eight patients had non-consistent results in 6-[^18^F]FDOPA PET and N1 assessment. As Table 4 shows in detail, this very heterogeneous group was composed of three patients with PS and five patients with Non-PS. Statistically, neither gender distribution, patient age, disease duration nor administered 6-[^18^F]FDOPA activity was significantly different to the patient pool with matched results (see Table 5). However, in 7 of these 8 patients, 6-[^18^F]FDOPA PET suggested a result in accordance with clinical findings. 6-[^18^F]FDOPA PET falsely classified just one patient suffering from a restless legs syndrome with a nigrostriatal dopamine deficit, whereas N1 evaluation showed a physiological swallow tail sign.

**Table 3:** Clinical diagnoses for different imaging result constellations

Imaging results	*n* (% of total)	PS	Non-PS
6-[^18^F]FDOPA abnormal/N1 abnormal	22 (51%)	22	0
6-[^18^F]FDOPA abnormal/N1 normal	4 (9%)	3	1
6-[^18^F]FDOPA normal/N1 abnormal	4 (9%)	0	4
6-[^18^F]FDOPA normal/N1 normal	13 (30%)	3	10

**Table 4:** Patient characteristics with mismatching results of 6-[^18^F]FDOPA and N1 evaluation

Patient no	6-[^18^F]FDOPA result	N1 result	Clinical diagnosis	Imaging modality with correct diagnosis	Sex	Age (years)	Duration of disease (years)	Activity (MBq)
1	Abnormal	Normal	RLS	N1	Female	76	1	211
2	Abnormal	Normal	PSP	6-[^18^F]FDOPA	Male	74	2	221
3	Normal	Abnormal	ET	6-[^18^F]FDOPA	Female	58	10	213
4	Normal	Abnormal	MND	6-[^18^F]FDOPA	Male	60	3	198
5	Abnormal	Normal	IPS	6-[^18^F]FDOPA	Male	65	2	181
6	Abnormal	Normal	PSP	6-[^18^F]FDOPA	Female	62	1	226
7	Normal	Abnormal	ET	6-[^18^F]FDOPA	Male	51	7	237
8	Normal	Abnormal	VP	6-[^18^F]FDOPA	Male	74	1	193

*RLS* restless legs syndrome, *PSP* progressive supranuclear palsy, *ET* essential tremor, *MND* motor neuron disease, *VP* vascular parkinsonism

**Table 5:** Patient characteristics depending from 6-[^18^F]FDOPA and N1 results

Characteristics	6-[^18^F]FDOPA abnormal/N1 abnormal	6-[^18^F]FDOPA normal/N1 normal	6-[^18^F]FDOPA abnormal/N1 normal	6-[^18^F]FDOPA normal/N1 abnormal	*p* value
No. of male/female patients	13/9	5/8	2/2	3/1	0.553
Age (years) mean ± SD (range)	62.09 ± 11.11 (38–82)	64.31 ± 15.96 (20–81)	69.25 ± 6.80 (62–76)	60.75 ± 9.64 (51–74)	0.714
Duration of disease (years) mean ± SD (range)	2.50 ± 2.06 (1–10)	4.38 ± 3.57 (1–10)	1.50 ± 0.58 (1–2)	5.25 ± 4.03 (1–10)	0.070
Activity (MBq) mean ± SD	215.32 ± 16.95	218.08 ± 21.08	209.75 ± 20.16	210.25 ± 19.76	0.820

Statistical results of multiple group comparison (ANOVA)

In summary, as compared with the clinical gold standard, 6-[^18^F]FDOPA PET had one false positive and two false negative results, whereas N1 had four false positive and five false negative results. Accordingly, PET had an excellent sensitivity of 92.6%, specificity of 93.8%, accuracy of 93.0%, positive predictive value of 96.2% and negative predictive value of 88.2%. In comparison, the sensitivity of N1 was 81.5%, specificity was 75.0%, accuracy was 79.1%, positive predictive value was 84.6% and negative predictive value was 70.6%.

Because of the wide range of disease duration until PET/MR imaging, we compared 6-[^18^F]FDOPA and N1 assessment in short (up to 2 years) and long (more than 2 years) intervals after onset of disease (see Table 6). For short and long intervals, 6-[^18^F]FDOPA accuracies were 96 and 88%, whereas N1 accuracies were 85 and 71%, respectively. Intermodality agreement was fair to good in short interval (*k* = 0.570, *p* = 0.003) and long interval scanning (*k* = 0.658, *p* = 0.004). In short interval scans, there were five 6-[^18^F]FDOPA-N1 discordant cases, of which four cases matched between 6-[^18^F]FDOPA findings and final diagnosis (see also Table 3). In long interval scans, all three mismatch cases where correctly diagnosed by 6-[^18^F]FDOPA PET alone.

**Table 6:** Imaging results, derived from short and long intervals between clinical onset of symptoms and PET/MR examination, and their corresponding clinical diagnoses

Short interval (≤ 2 years)	Imaging modality	Imaging result abnormal	Imaging result normal	Number of patients (total)	Clinical diagnosis
	6-[^18^F]FDOPA PET	12	0	12	IPS
		6	0	6	APS
		1	7	8	Non-PS
	N1	11	1	12	IPS
		4	2	6	APS
		1	7	8	Non-PS

Long interval (> 2 years)	Imaging modality	Imaging result abnormal	Imaging result normal	Number of patients (total)	Clinical diagnosis
	6-[^18^F]FDOPA PET	6	0	6	IPS
		1	2	3	APS
		0	8	8	Non-PS
	N1	6	0	6	IPS
		1	2	3	APS
		3	5	8	Non-PS

Besides the visual interpretation, 6-[^18^F]FDOPA PET allows semi-quantitative assessment of dopamine metabolism in the basal ganglia. 6-[^18^F]FDOPA uptake ratios of the caudate and the putamen on both sides to the occipital region were significantly lower in PS patients than in Non-PS patients (e.g., 2.11 ± 0.29 vs. 2.91 ± 0.36 in the right putamen, *n* < 0.001), further substantiating the qualitative results. For a possibly better differentiation between idiopathic and atypical parkinsonian syndromes, striatal left–right and caudal-rostral gradients were examined. Mean uptake ratio of each left and right putamen to the occipital region did not differ significantly between IPS and APS patients. A profound caudal-rostral gradient was observed in the left and right putamen of both groups: e.g., in IPS patients, mean standardized uptake value (SUV) maximum in the right anterior vs. posterior putamen was 3.01 ± 0.38 vs. 2.37 ± 0.42, respectively (*p* < 0.001). However, caudal-rostral gradients of both hemispheres were statistically similar in patients with IPS and APS.

### Secondary analysis

A normal PET imaging result constitutes an exclusion criterion for the diagnosis of PD by the MDS criteria [8]. In a secondary analysis (see Fig. 3), we therefore excluded four patients with normal 6-[^18^F]FDOPA PET in which the result of PET imaging changed the diagnosis based on the hospital charts. All four patients had clinically suspected IPS prior to PET. After PET imaging, diagnosis changed to VP in two patients, to ET in one patient and to AD in the last patient, respectively. With the remaining 39 patients, the values obtained for sensitivity, specificity and intermodality agreement of 6-[^18^F]FDOPA PET and N1 assessment did not change significantly, which is shown in Table 7.

**Table 7:** Statistical comparison of primary analysis with a consecutive secondary analysis, in which four patients were excluded because of normal PET results that changed the clinical diagnosis from IPS to Non-PS

	Primary analysis (43 patients)		Secondary analysis (39 patients)	
Imaging modality	6-[^18^F]FDOPA PET	N1 (%)	6-[^18^F]FDOPA PET	N1 (%)
Sensitivity	92.6%	81.5	92.6%	81.5
Specificity	93.8%	75.0	91.7%	75.0
Positive predictive value	96.2%	84.6	96.2%	88.0
Negative predictive value	88.2%	70.6	84.6%	64.3
Accuracy	93.0%	79.1	92.3%	79.5
Kappa (all patients)	0.611 (*p* < 0.001)		0.604 (*p* < 0.001)	

## Discussion

In the present study, we found a high accuracy of 6-[^18^F]FDOPA and only a moderate accuracy of N1 evaluation in discriminating PS from Non-PS.

6-[^18^F]FDOPA is a well-established PET tracer that illustrates terminal dopa decarboxylase activity, providing a valuable tool for the identification of early PD [3]. The high sensitivity, specificity and accuracy in our study go in line with other studies, who reported sensitivities and specificities of 90–100% and 91–100%, respectively [19, 20]. However, presynaptic dopaminergic function is known to be impaired not only in IPS, but also in APS, thus resulting in a low specificity for discriminating both of them [21, 22]. This was underlined by the results of our semi-quantitative metabolism analysis, which revealed no substantial differences in striatal 6-[^18^F]FDOPA uptake between both entities, but a profound caudal-rostral gradient in the putamen on both hemispheres in IPS and APS. To address this issue, further studies regarding specific 6-[^18^F]FDOPA uptake patterns in the striatum are still required.

Nigrosome 1 is the largest cluster of dopaminergic cells in the substantia nigra pars compacta and can be visualized by high-resolution SWI MRI. In IPS patients, the otherwise physiological swallow tail appearance of the N1 is lost, which indicates nigral degeneration [23]. Schwarz et al. reported almost perfect sensitivity, specificity and accuracy of 95% [7] for N1 assessment to discriminate IPS patients from healthy controls. A recent meta-analysis confirmed the excellent diagnostic accuracy of N1 assessment [24]. However, in our study, N1 evaluation had a moderate sensitivity, specificity and accuracy in discriminating PS from parkinsonism without dopaminergic deficit and was inferior to 6-[^18^F]FDOPA. Those discrepancies are due to completely different patient cohorts: our study cohort comprised only of patients with movement disorders of initially unknown origin that were recruited from our highly specialized ambulatory movement disorders clinic of the department of neurology in a university setting. No healthy controls were examined, which somewhat impairs comparability to earlier studies. In the general population, the IPS to APS ratio and the PS to Non-PS ratio are very dissimilar to our study cohort. A case–control study from Perez Akly et al. compared N1 evaluation results of 16 patients with IPS and 16 patients with essential tremor [25]. The sensitivity and specificity was 94% and 75–88%, respectively, which is higher than reported in our data (83% and 66%, respectively). In our study cohort, 2 of 6 patients with ET showed a loss of N1 signal. A possible explanation to this phenomenon might be the relatively long disease duration of 7 and 10 years, respectively, that could have led to nigral cell damage without significant involvement of dopaminergic cells, thus producing false-positive results.

Intermodality agreement of 6-[^18^F]FDOPA and N1 evaluation was very good in patients with clinical IPS, but declined in patients with APS and non-parkinsonian movement disorders. The main reasons for this may be the different principle of depicting the nigrostriatal system, alongside with our highly preselected cohort of patients. While there are several 6-[^18^F]FDOPA studies that describe qualitative and quantitative ways to discriminate between IPS and APS, such as unilateral uptake loss in the posterior putamen [26] or the presence of a rostro-caudal uptake gradient [27], the N1 assessment on 3 T MRI in daily routine happens solely on a visual basis. To enhance the potential of N1 imaging, quantification methods, like quantitative susceptibility mapping combined with histogram analysis, may be promising in the future [28].

In almost every case of disagreement between both imaging modalities, 6-[^18^F]FDOPA would have suggested the correct diagnosis, according to the clinical gold standard. The pool of patients with a mismatch result was very heterogenous and did not differ significantly from the matching group of 35 patients in terms of clinical diagnosis, age, duration of disease, etc.

Our study patients presented to the PET/MR center with a wide range of disease durations. In order to address this issue, we compared imaging results of subjects that were scanned within short (≤ 2 years) or long (≥ 2 years) intervals after symptom onset. In both intervals, overall sensitivity, specificity and accuracy of 6-[^18^F]FDOPA PET appeared to be higher than that of N1 imaging. Performances of both modalities declined slightly with longer duration of disease, but remained to be fair in terms of intermodality agreement. The number of false-positive and false-negative cases was too small to postulate any significant differences between short and long scanning intervals. However, for any scanning time point after symptom onset, IPS identification accuracy was excellent in both 6-[^18^F]FDOPA PET and N1 imaging. This finding goes in line with an earlier study of Stezin et al., who reported high sensitivity, specificity and accuracy of N1 imaging in PD patients, compared to healthy controls, with 5.2 ± 3.9 years mean duration of disease [29].

Our patient cohort included several disease entities, which can alternatively be divided into synucleopathies, like IPS and MSA, or tauopathies, such as PSP, CBD and AD, respectively. It is known that presynaptic dopaminergic imaging of synucleopathies and tauopathies may have different regional patterns of metabolism. Our study results may indicate a potential advantage of N1 and 6-[^18^F]FDOPA PET accuracy in synucleopathies, but, due to the small sample size, this statement remains speculative. Furthermore, there are several studies that do not show any differences between detection rate of synucleopathies or tauopathies in 3 T SWI MR imaging [30–33].

This study has several limitations. First, the retrospective study design, which bears a possible bias in our highly preselected patient cohort. Second, a relatively small sample size per particular diagnosis, because of missing clinical information for many patients. Third, the definition of clinical diagnosis as the gold standard. The sensitivity and specificity of the clinical diagnosis are 88% and 68%, respectively, as confirmed by post-mortem findings in patients with IPS [34]. Although the clinical follow-up was at least six months in all of our patients, the results of 6-[^18^F]FDOPA PET and N1 evaluation could have influenced the final diagnosis, because our neurological specialists were not blinded to the scan results. To address this bias, we performed a secondary analysis, where four patients were excluded because their former clinical diagnosis of IPS had to be corrected to Non-PS after normal PET results, according to MDS diagnostic criteria. Overall statistical performances of both imaging modalities did not change significantly to our primary analysis. This may indicate that the superior performance of PET imaging is not a consequence of their inclusion in the MDS diagnostic criteria.

Our divergent imaging results of patients with APS could be explainable by neuropathological considerations: dopaminergic neurons are thought to degenerate by a "dying back" mechanism, i.e., the extent of terminal loss in the striatum is generally larger than the extent of neuron loss in the substantia nigra [35]. This could account for higher sensitivity and specificity of 6-[^18^F]FDOPA PET than that of N1 imaging. Yet, the relationship between imaging findings and neuropathology is not static. It is unclear what the 6-[^18^F]FDOPA PET abnormalities in APS mean on a patho-anatomical level, and whether N1 abnormalities really reflect neurodegeneration or other processes like glial reactions.

However, as presynaptic dopaminergic imaging is an established and reliable tool for assessing parkinsonism, its outcomes might be generally better accepted by clinicians, thus resulting in another possible bias towards it. To finally overcome these uncertainties, histopathological examination might be the only way, which is not feasible in clinical studies.

In summary, long-term prospective studies with a higher number of patients and healthy controls are required to confirm the findings of the present study and to assess disease progression and severity.

## Conclusion

In our highly preselected group of patients with parkinsonism, 6-[^18^F]FDOPA PET and N1 evaluation had an overall good intermodality agreement. The diagnostic agreement between 6-[^18^F]FDOPA PET and N1 evaluation was very good in cases of clinically suspected idiopathic Parkinson syndrome and fair in atypical parkinsonian syndromes, but poor in patients with non-parkinsonian disorders.

6-[^18^F]FDOPA showed higher sensitivity, specificity and accuracy in discriminating parkinsonian syndromes from non-parkinsonian disorders than the N1 evaluation. In mismatching results, 6-[^18^F]FDOPA PET correlated much more frequently with the clinical diagnosis than N1.

In summary, the additional benefit of N1 assessment in patients with APS or parkinsonism without dopaminergic deficit still needs to be proven by prospective studies.

## Data Availability

The data that support the findings of this study are available from the corresponding author upon reasonable request.

## Abbreviations

FDOPA: Fluorodopa
PET: Positron emission tomography
SWI: Susceptibility-weighted imaging
MRI: Magnetic resonance imaging
N1: Nigrosome 1
STS: Swallow tail sign
IPS: Idiopathic Parkinson syndrome
APS: Atypical parkinsonian syndrome
PSP: Progressive supranuclear palsy
MSA: Multiple system atrophy
CBD: Corticobasal degeneration
LBD: Dementia with Lewy Bodies
PS: Parkinson syndrome
MDS: Movement Disorder Society
VOI: Volume of interest
ET: Essential tremor
DIP: Drug-induced parkinsonism
VP: Vascular parkinsonism
RLS: Restless legs syndrome
MND: Motor neuron disease
AD: Alzheimer’s disease
HD: Huntington’s disease
SUV: Standardized uptake value
